# The effect of the nonselective TNF inhibitor etanercept and of the selective TNF inhibitor XPro1595 on lesioned supraspinatus muscle

**DOI:** 10.1042/BSR20253559

**Published:** 2025-12-08

**Authors:** Christopher Aboo, Kate Lykke Lambertsen, Sohail Nasseri, Ming Ding, Peter Toft Jensen, Thi My Linh Ta, Nicholas Ditzel, Henrik Daa Schrøder, Allan Stensballe, Eva Kildall Hejbøl, Lars Henrik Frich

**Affiliations:** 1 Department of Health Science and Technology, Aalborg University, Aalborg, Denmark; 2 Sino-Danish Center for Research and Education, University of Chinese Academy of Sciences, Beijing, China; 3 Department of Neurobiology, Institute of Molecular Medicine, University of Southern Denmark, Odense, Denmark; 4 Department of Neurology, Odense University Hospital, Odense, Denmark; 5 BRIDGE – Brain Research – Inter-Disciplinary Guided Excellence, Department of Clinical Research, University of Southern Denmark, Odense, Denmark; 6 Orthopaedic Research Laboratory, Department of Orthopaedic Surgery & Traumatology, Odense University Hospital, Odense, Denmark; 7 Department of Clinical Research, University of Southern Denmark, Odense, Denmark; 8 KMEB, Molecular Endocrinology, Odense University Hospital, Odense, Denmark; 9 Winsløw Unit for Anatomy, Histology and Plastination, Department of Molecular Medicine, University of Southern Denmark, Odense, Denmark.; 10 Clinical Cancer Research Center, Aalborg University Hospital, Aalborg, Denmark; 11 Department of Orthopaedics, Hospital of Southern Jutland, Aabenraa, Denmark; 12 Department of Regional Health Research, University of Southern Denmark, Odense, Denmark

**Keywords:** cytokines, inhibition, mitochondria, regeneration, skeletal muscle, tumour necrosis factors

## Abstract

The cytokine tumor necrosis factor (TNF), a major regulator of inflammatory responses, exists in both a membrane-bound form and a soluble form. We used the nonselective TNF inhibitor etanercept and the selective inhibitor XPro1595 and compared supraspinatus muscle cytokine levels, histology, and proteomic signatures in mice after supraspinatus tendon tear. The aim was to investigate the effect of anti-TNF treatment on the early inflammatory response in the muscle after tendon tear. In addition, the effect on body composition and bone mineral content was compared in naive mice after 2 months of treatment with either etanercept or XPro1595 using dual-energy X-ray absorptiometry (DEXA) and micro-CT. Inhibition of TNF did not significantly affect DEXA indexes of body composition nor bone microarchitecture, apart from increased structure model index and decreased bone surface density at 14 days, and bone surface to volume ratio at 2 months. Supraspinatus tendon tear caused extensive inflammatory changes in the supraspinatus muscle and initiated a regenerative response. However, TNF inhibition did not significantly affect these processes recorded as density in the lesioned supraspinatus muscle of macrophages and myogenin-positive nuclei. Although both inhibitors had an effect on mitochondrial proteins, particularly etanercept tended to modulate mitochondrial function, and eternacept also influenced NF-κB signaling. Modulation of the mitochondrial proteome and the influence on NF-κB signaling seen after etanercept treatment could correspond with its known effect on apoptosis.

## Introduction

Tumor necrosis factor (TNF) is an immunomodulatory cytokine that plays a critical role in the regulation of immune responses, particularly in chronic inflammatory conditions such as rheumatoid arthritis, inflammatory bowel disease, and muscular dystrophies [1,2] . While TNF is essential for host defense and tissue homeostasis, excessive or prolonged TNF activity has been implicated in pathological muscle wasting, weakness, and metabolic dysfunction [3,4]. Given these detrimental effects, anti-TNF therapies have emerged as a potential strategy to mitigate inflammation-associated skeletal muscle deterioration, particularly in the context of rotator cuff (RC) injuries.

The supraspinatus muscle, a key component of the RC, is highly susceptible to atrophy and fatty infiltration following RC tendon tears, leading to impaired shoulder function and poor surgical outcomes [5,6]. Inflammation in the RC muscles is a part of the response to the tendon lesions [7], and the muscle damage is thought to hamper shoulder rehabilitation after reconstructive tendon surgery. Inflammatory pathways, including TNF signaling, are thought to contribute significantly to muscle degeneration and failed muscle regeneration post-injury [8]. Anti-TNF agents, including monoclonal antibodies (e.g. infliximab, adalimumab) and soluble TNF receptors (e.g. etanercept), have been widely used in the treatment of autoimmune and inflammatory diseases and may offer a therapeutic avenue for preserving supraspinatus muscle integrity after RC tears [9].

Our hypothesis is that TNF inhibition may attenuate muscle atrophy, reduce fibrosis, and promote muscle regeneration in the setting of RC injuries. However, the precise mechanisms by which anti-TNF therapies influence supraspinatus muscle recovery remain incompletely understood. Additionally, the therapeutic potential of TNF inhibition in improving postoperative outcomes and functional recovery after RC repair warrants further investigation [10].

Etanercept, a human dimeric TNF receptor fusion protein, blocks both the membrane-bound form of TNF and the soluble form of TNF (solTNF) in the extracellular compartment [11]. However, as with other anticytokine therapies, etanercept weakens the immune defense against infection. Anti-TNF therapy may also cause weight gain [12] and changes in body composition [13].

XPro1595, also called pegipanermin or XPro, is a protein drug designed to bind to and inhibit a solTNF. In contrast to etanercept, this selective TNF inhibitor targets only solTNF, which is then thought to decrease the risk of infections compared with etanercept.

In order to develop a therapeutic strategy for the treatment of traumatic muscle injuries, we examined the impact of treatment with a conventional non-selective TNF inhibitor (etanercept) and a selective solTNF inhibitor (XPro1595) [14] in a supraspinatus tendon tear mice model. As an outcome, we analyzed the cytokine levels, proteomic profile, and histological changes of the supraspinatus muscle.

## Materials and methods

### Mice

Young adult (7–8 weeks) C57BL/6 mice were purchased from Taconic Ltd. (Ry, Denmark) and transferred to the Laboratory of Biomedicine, University of Southern Denmark, where they were allowed to acclimatize for 1–2 weeks before surgery and pharmacological treatment. Animals were housed under diurnal lighting conditions and given free access to food and water. All experimental protocols were approved by The Danish Animal Inspectorate under the Ministry of Food and Agriculture (J. No. 2016−15−0201–00854) and all efforts were made to minimize pain and distress. Naïve mice included in this study and allowed 2 months of survival after treatment were also part of a previous study on the effect of TNF treatment on cognitive functions [15].

### Pharmacological treatment

Etanercept (Enbrel, Amgen-Wyeth, Thousand Oaks, CA) or XPro1595 (Xencor Inc, Monrovia, CA) [14] was administered subcutaneously (s.c.) at a dose of 10 mg/kg every third day as previously described in Yli-Karjanmaa et al. [15]. Saline was used as the vehicle and also served as a placebo control. In the RC tear model, treatment was given immediately after surgery and again after three days.

### Group size and study design

Mice that underwent experimental supraspinatus tendon tear were killed five days after surgery, as this is the time point at which we previously identified the peak of post-surgical inflammation in the supraspinatus muscle in our RC tendon tear model [16] . Mice were weighed prior to surgery and on day 5, and weight changes were calculated as Δweight (day 5 – day 0). Post-surgical mortality was 5%.

Groups of mice for micro-CT analysis consisted of male mice treated s.c. for 14 days (*n* = 10/group) or 2 months (*n* = 8/group) (Table 1).

**Table 1:** Experimental groups. SS: supraspinatus, m: male, f: female. Time of euthanasia indicates the days between the start of treatment to cessation of life

Surgery	Treatment	Number of mice/sex	Analyses	Time of euthanasia
SS tendon tear	XPro1595	6/f	Proteomics, chemiluminescence, and grip strength	5 days
SS tendon tear	Etanercept	7/f	Proteomics, chemiluminescence, and grip strength	5 days
SS tendon tear	Saline	6/f	Proteomics, chemiluminescence, and grip strength	5 days
SS tendon tear	XPro1595	5/m	Histology	5 days
SS tendon tear	Etanercept	5/m	Histology	5 days
SS tendon tear	Saline	5/m	Histology	5 days
No	XPro1595	10/m	Micro-CT	14 days
No	Etanercept	10/m	Micro-CT	14 days
No	Saline	10/m	Micro-CT	14 days
No	XPro1595	8/m	Micro-CT and DEXA scan	2 months
No	Etanercept	8/m	Micro-CT and DEXA scan	2 months
No	Saline	8/m	Micro-CT and DEXA scan	2 months

### Supraspinatus tendon tear model

Mice were anesthetized using a ketamine (100 mg/kg, VEDCO Inc) and xylazine (10 mg/kg, VEDCO Inc) cocktail injected intraperitoneally (i.p.) and positioned in a left lateral decubitus position on a heating pad under a surgical microscope. As described previously [16], an incision was placed just above the elbow and directed posteriorly and inferiorly toward the ear. The deltoid was identified and released from the acromion with sharp dissecting scissors. The proximal humerus was grasped for stability and externally rotated to visualize the coracoacromial arch. To visualize the supraspinatus tendon, blunt dissection was used between the humeral head and the coracoacromial arch. The supraspinatus tendon was sharply released using scissors, allowing the supraspinatus muscle to retract. The deltoid was reflected, and the skin incision closed using 7–0 sutures. Mice were then injected with saline to prevent dehydration and given buprenorphine hydrochloride (0.001 mg/20 g body weight Temgesic) three times at 8-h intervals for postsurgical analgesia. Mice were housed separately in a recovery room and monitored for a 24-to-48-h recovery period. Thereafter, mice were returned to the conventional animal facility.

### Grip strength

Grip strength was measured before and five days after surgery using a grip strength meter (BIO-GT-3, BIOSEB, Vitrolles, France) as previously described [17]. We measured the peak force generated when the mouse loosened its grip when being gently pulled horizontally backwards. The two front legs were tested individually. The peak force of five trials was recorded, and the highest score was registered. Mice were excluded from the test if they could not complete the trial.

### Histology

Mice designated for immunohistochemistry consisted of male mice subjected to experimental supraspinatus tendon tear in their left shoulder. These mice were assigned to receive either etanercept (*n* = 5), XPro1595 (*n* = 5) or served as controls and were administered saline (*n* = 6) on the day of the tear and three days later. Euthanasia was performed by cervical dislocation five days after the tear.

All supraspinatus muscles were fixed in 4% paraformaldehyde (PFA) for paraffin embedment. Immunohistochemistry was performed on 2-µm-thick, dewaxed tissue sections with EnVision+ Single Reagent (horseradish peroxidase (HRP), rabbit, Dako-Agilent) for Iba1 (Wako Pure Chemical Industries, polyclonal rabbit, 1:1000), CD3 (LabVision - NeoMarkers, SP7, rabbit, 1:200) and myogenin (Cell Marque, EP162, rabbit, 1:100). For F4/80 (Bio-Rad, rat, Cl:A3-1, 1:100), we used rabbit-anti-rat IgG (Abcam, ab102248) and EnVision+ System–HRP Labelled Polymer Anti-Rabbit (Dako-Agilent). Epitope retrieval was done by boiling in a microwave oven for 15 min in Tris-EGTA buffer or Target Retrieval Solution (Agilent).

For each muscle, two parallel sections 0.5 mm apart were analyzed. The density of Iba1 and F4/80 positive cells was determined by counting three fields of 1 mm^2^ in each muscle section: one at the lateral (tendon near) end, one medially, and one between the two (in the middle of the muscle). For CD3 and myogenin, the density of positive cells or nuclei was determined in the full muscle sections.

Immunofluorescence for mitochondria was performed with Tom20 (FL-145) rabbit polyclonal antibody (1:1000) and DAPI (Merck Sigma-Aldrich, D9542). Epitope retrieval was done at 100°C for 30 min in 10 mM citrate buffer; pH 6 with 0.05% Tween. The sections were then treated with autofluorescence Eliminator Reagent (Merck-Millipore, Temecula, CA) and incubated with the primary antibody at 4°C overnight. As a secondary antibody, Alexa-594-conjugated goat-anti-rabbit IgG (Life Technologies, CA, 1:500) was used. The volume fraction of TOM^+^ mitochondria was estimated by analyzing 2–10 images from an Olympus DP70 digital camera at 40× magnification from each muscle. To reduce differences in fluorescence intensity caused by bleaching during the capture of images, the background intensity of muscle fibers was normalized so only subsarcolemmal mitochondria were visible. Using ImageJ [18], a standardized threshold was applied to red channel images to acquire relative values for volume fractions of mitochondria.

### Body composition measurements

Body composition was examined prior to pharmacological treatment and 2 months after treatment on fully sedated (1.5% isoflurane anesthesia) mice using non-invasive dual-energy X-ray absorptiometry (DEXA) (PIXImus 2, Version 1.44, GE Lunar, Madison, WI), as previously described [19]. Measurements included total tissue mass (g), fat %, lean tissue mass (g), lean %, bone area (cm^2^), bone mineral content (BMC, g), and bone mineral density (BMD, g/cm^2^).

### Micro-CT scanning and microarchitectural analysis

Mice were killed 14 days or 2 months after pharmacological treatment using an overdose of pentobarbital (200 mg/ml) containing lidocaine (20 mg/ml) (Glostrup Apotek, Denmark) and perfused through the left ventricle using ice-cold 4% PFA. The left humeri were dissected and stored in 4% PFA.

To quantify 3D microarchitectural properties, all the left humeral bone specimens were micro-CT scanned with a high-resolution scanner (vivaCT 40, Scanco Medical AG, Brüttisellen, Switzerland). The scanned images resulted in 3D reconstruction cubic voxel sizes of 10.5*10.5*10.5 µm^3^ (2048*2048*2048 pixels) with 32-bit gray levels. To obtain accurate 3D imaging datasets, the micro-CT images were segmented by applying the segmentation techniques previously described [20].

The region of interest of each cancellous bone was defined and carefully contoured from accurate image data. 3D microarchitectural properties of the cancellous bone were calculated by means of true, unbiased, and assumption-free 3D methods. Cancellous bone volume fraction (BV/TV, %), structure model index (SMI, -), trabecular thickness (TbTh, µm), degree of anisotropy (DA, -), connectivity density (CD, mm^-3^), bone surface density (BS/TV, mm^-1^), bone surface to volume ratio (BS/BV, mm^-1^), trabecular separation (TbSp, µm), and trabecular number (TbN, mm^-1^) were calculated [21].

### Sample collection for chemiluminescence and proteomics

The lesioned and non-lesioned supraspinatus muscles from mice that were killed by cervical dislocation were dissected, split in two, frozen with dry ice, and stored at -80°C until further processing.

### Chemiluminescence analysis

Supraspinatus muscle tissue was lysed in complete mesoscale buffer (150 mM NaCl, 20 mM Tris, 1 mM EDTA, 1 mM EGTA, 1% Triton X-100; pH 7.5) containing Phosphatase Inhibitor Cocktail 2 and 3 (Sigma-Aldrich) and Complete Mini EDTA-free Protease inhibitor (Roche) and rotated end-over-end for 1 h at 4°C. Samples were centrifuged at 13,000 rpm for 20 min, 4°C. Protein concentrations were determined according to the protocol from the Micro BCA protein assay kit (Thermo Fisher Scientific). XPro1595 levels in supraspinatus muscles were estimated using a human TNF V-Plex immunoassay (Mesoscale Discovery) as previously described [17]. The standard in the kit was replaced with XPro1595, which was diluted in kit diluent number 2. Etanercept was measured using a human TNFRII Ultra-Sensitive immunoassay (Mesoscale Discovery) [17]. The standard in the kit was replaced with etanercept diluted in the kit diluent number 2. TNF, interleukin (IL)-1β, IL-6, CXCL1, IL-10, IL-12p70, IL-2, IL-4, IL-5, and interferon-gamma (IFN-γ) levels were estimated using a Mouse Proinflammatory V-Plex Plus Kit, and TNFR1 and TNFR2 levels were estimated using mouse TNFRI or TNFRII Ultra-sensitive Kits (all Mesoscale). Kits were read on a SECTOR Imager 6000 plate reader (Mesoscale Discovery) according to the manufacturer’s instructions. All samples were run in duplex, and coefficient of variation values below 25% were accepted. The lower limit of detection (LLOD) was a calculated concentration based on a signal 2.5 standard deviation (SD) above the blank (zero) calibrator. When protein levels were below LLOD, a value equal to half the LLOD was assigned for statistical analysis. LLOD values for each protein were as follows: IFN-γ = 0.04 pg/ml, IL-10 = 0.95 pg/ml, IL-12p70 = 9.95 pg/ml, IL-1β = 0.11 pg/ml, IL-2 = 0.22 pg/ml, IL-4 = 0.14 pg/ml, IL-5 = 0.07 pg/ml, CXCL1 = 0.24 pg/ml, TNF = 0.13 pg/ml, IL-6 = 0.61 pg/ml, TNFR1 = 0.98 pg/ml, and TNFR2 = 9.43 pg/ml.

### Homogenization and preparation of tissue prior to proteomic analysis

Supraspinatus muscle tissue was prepared for liquid chromatography (LC) coupled mass spectrometry (MS) analysis using a filter-aided protein digestion protocol. Samples were weighed and transferred to 1.5 ml impact-resistant tubes containing 500 μl lysis buffer and half a spoonful of lysing beads, followed by homogenization for 10 min using a cooled bullet blender (Next Advance BB24-AU). Foam was removed by centrifugation, and proteins were denatured at 95℃ for 5 min. Protein concentration of each sample was then determined by A280 NanoDrop 1000 UV spectrophotometry (Thermo Scientific, Waltham, MA).

Tissue lysate volume containing 100 μg protein was transferred to a 30-kDa YM-10 tilted spin filter, and 200 μl of lysis buffer was added. The spin filters were then centrifuged at 14,000 g until ∼ 50 μl was left in the spin filter cup. A volume of 200 μl of lysis buffer was then added again and centrifugation repeated. A volume of 200 μl of digestion buffer was then added to a final volume of 300 μl followed by reduction and alkylation with 1:50 vol of tris(2-carboxy-ethyl)phosphine hydrochloride 0.5 M and 1:10 vol chloroacetamide 0.5 M in triethylammonium bicarbonate buffer, vortexed, incubated for 30 min at 37℃ and washed with 200 μl digestion buffer followed by centrifugation. Proteins were then tryptically digested using 200 μl digestion buffer containing 2 µg trypsin in new collection tubes and incubated overnight at 37℃ in a humid environment.

The resulting peptides were recovered by centrifugation, and the digestion buffer was removed using phase separation. Briefly, 1500 μl of ethyl acetate and 20 μl trifluoroacetic acid (TFA) were added, followed by vortexing, centrifugation for 1 min and removal of the top phase by pipetting. The process was repeated, and the lower phase containing the clean peptides was dried in a vacuum centrifuge and stored at -80℃ until analysis.

### LC-MS analysis

Peptides were resuspended in reversed phase loading buffer containing 2% acetonitrile (ACN), 0.1% formic acid (FA), and 0.1% TFA prior to LC-MS analysis. Samples were then vortexed and ultrasonicated for 1 min, followed by centrifugation for 1 min at 14,000 g. Samples were analyzed by a Dionex RSLC Proflow nanoUPLC system, connected to a Bruker timsTOF mass spectrometer (Bruker, Bremen, Germany). For ionization, a NanoSpray ion source (Proxeon, Odense, Denmark) was used. The flow was 200 nl per min for sample loading onto a trapping column (Acclaim PepMap100 C18 2 cm, 100 μm ID; 5 μm particle size column, Thermo Scientific). The flow rate of the nanoflow was 200 nl/min for the peptide separation on the analytical column, a 75 cm Acclaim Pepmap RSLC C-18, 75μ ID column connected with nanovipers. The Picotip ‘Silicatip’ emitter from New Objective was used for nano-electrospray. The buffers of the liquid chromatography system consisted of buffer A (0.1% FA) and buffer B (99.9% ACN, 0.1% FA). The gradient used was 5–35% buffer B over 88 min. The mass spectrometer system was operated in data-dependent acquisition mode. Mass range of m/z 350 to 2200 was acquired at a resolution of approximately 45,000 for each full scan. The mass spectrometer would acquire up to 2 MS/MS acquisitions on abundant peptide precursor ions in each cycle. Precursor ions were isolated by employing a quadrupole isolation window of 2.0 m/z and fragmented in a collision-induced dissociation collision cell dynamic collision energy, optimized for peptide fragmentation. Dynamic exclusion was set to 30 s.

Raw output data were identified using MaxQuant version 1.6.0.1 with the Andromeda search engine [22]. The raw data were searched against a reference proteome of Mus musculus (Proteome ID UP000000589, 16,854 entries) from the UniprotKB database. As a cutoff, a false discovery rate of 0.1% was used for both the protein and peptide level with the ‘match between runs’ feature enabled. Fixed modification was set to carbamidomethylation. N-terminal acetylation and oxidation of methionine were set as variable modifications. The mass spectrometry proteomics data have been deposited to the ProteomeXchange Consortium via the PRIDE (ref PubMed ID: 34723319) partner repository with the dataset identifier PXD035331.

### Proteomics data processing

Using Perseus (v 1.6.10) [22], the data were log2-transformed, reverse hits, potential contaminants, and proteins whose identification was based on less than two peptides were removed. Proteins that were present in less than 50% of the samples within at least one of the treatment groups were also removed. The replicates were then combined by taking the mean, and missing values were imputed from a normal distribution using default Perseus parameters to simulate signals from low-abundant proteins. The resulting data matrix was imported into R (v 3.6.1) using PerseusR (v 0.3.4) [23].

### Bioinformatics analysis

To test if samples clustered according to lesioned and non-lesioned sites based on their proteomic profile, a multilevel principal component analysis (PCA) was adapted from the MixOmics package (version 6.8.5) using the PCA function [24]. The associated MixOmics plot functions were then used to create a PCA score plot to visualize the natural discrimination of samples based on their proteomic and cytokine signatures and a loading plot to display the most important proteins for driving the discrimination. A PCA model was also made using only the lesioned samples to see if one could classify proteomes according to their respective group without prior knowledge about the treatment. Finally, a PCA model was made to assess if mice clustered naturally based on their bone microarchitecture measures following 14 days or 2 months of treatment with etanercept, Xpro1595, or saline.

A sparse partial least squares–discriminant analysis (sPLS–DA) was also adapted from the MixOmics R package to identify proteins that could discriminate between the three treatment groups. The sPLS–DA is a supervised model that can identify the most discriminative variables for classifying the proteomes according to treatment [24]. The optimal number of components and variables on each component was determined by evaluating the performance of the model using the MixOmics perf and tune.splsda functions with a three-fold cross-validation and 200 repetitions. The best performance of the sPLS–DA model (lowest classification error rate) was achieved using two components with 5 and 21 proteins on component one and two, respectively.

A score plot was made to illustrate if the sPLS–DA model was able to discriminate between samples based on the selected variables, and the cim function was used to create heat maps based on hierarchical clustering using the selected variables from the sPLS–DA [24].

### Statistical analysis

For analysis of DEXA results, repeated measures two-way ANOVA (analysis of varance) was used. For micro-CT, one-way ANOVA with Tukey’s post hoc was used. For the grip strength test results, we used the Student’s t-test and Kruskal–Wallis. For cytokine levels, one-way ANOVA was used, and for histology results, we used two-way ANOVA and the Kruskal–Wallis test.

## Results

To address possible side effects of TNF inhibition on body mass and bone structure, we examined a series of treated and non-treated mice for body composition and bone mineral content with DEXA and micro-CT, respectively.

### TNF inhibition does not affect body composition or bone microstructure

**Figure 1:**
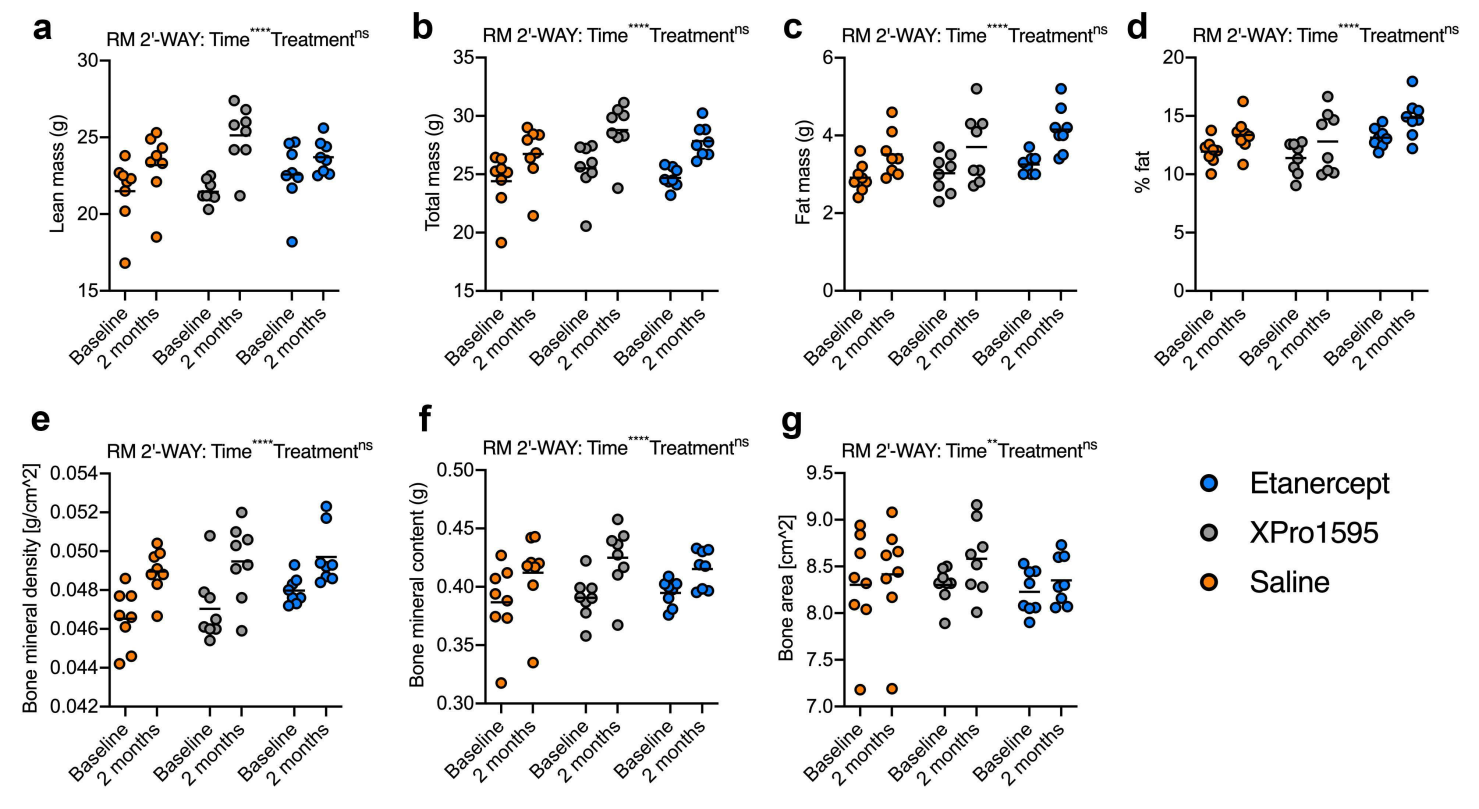
Long-term effect on body composition of anti-TNF treatment. DEXA scan demonstrated (**a**) Lean mass increased over time with no differences between groups (Time ****P < 0.0001, F1,21 = 30.00; Treatment ns; interaction *P = 0.04, F2,21 = 3.76). (**b**) Total mass increased over time with no differences between groups (Time ****P < 0.0001, F1,21 = 318.0; Treatment ns; interaction ns). (**c**) Fat mass increased over time with no differences between groups (Time ****P < 0.0001, F1,21 = 37.23; Treatment ns; interaction ns). (**d**) The percentage (%) of fat increased over time with no differences between groups (Time ****P < 0.0001, F1,21 = 28.74; Treatment ns; interaction ns). (**e**) Bone mineral density increased over time with no differences between groups (Time ****P < 0.0001, F1,21 = 78.65; Treatment ns; interaction ns). (**f**) Bone mineral content increased over time with no differences between groups (Time ****P < 0.0001, F1,21 = 63.37; Treatment ns; interaction ns). (**g**) Bone area increased over time with no differences between groups (Time ****P < 0.0001, F1,21 = 9.57; Treatment ns; interaction ns). repeated measures two-way ANOVA, data are presented as mean ± SD with *n* = 8/group.

Initially, we assessed whether XPro1595 or etanercept affected body composition in healthy C57Bl/6 mice using DEXA scan (Figure 1). We tested whether treatment with s.c. injections of 10 mg/kg XPro1595 or 10 mg/kg etanercept every third day for 2 months affected lean mass (g) (Figure 1a), total mass (g) (Figure 1b), fat mass (g) (Figure 1c), % fat (Figure 1d), BMD (g/cm^2^) (Figure 1e), BMC (g) (Figure 1f), or bone area (cm^2^) (Figure 1g). Even though we observed changes over time in all parameters tested, we did not observe any differences between groups, suggesting that 2 months of s.c. XPro1595 and etanercept treatment does not affect body composition.

**Figure 2:**
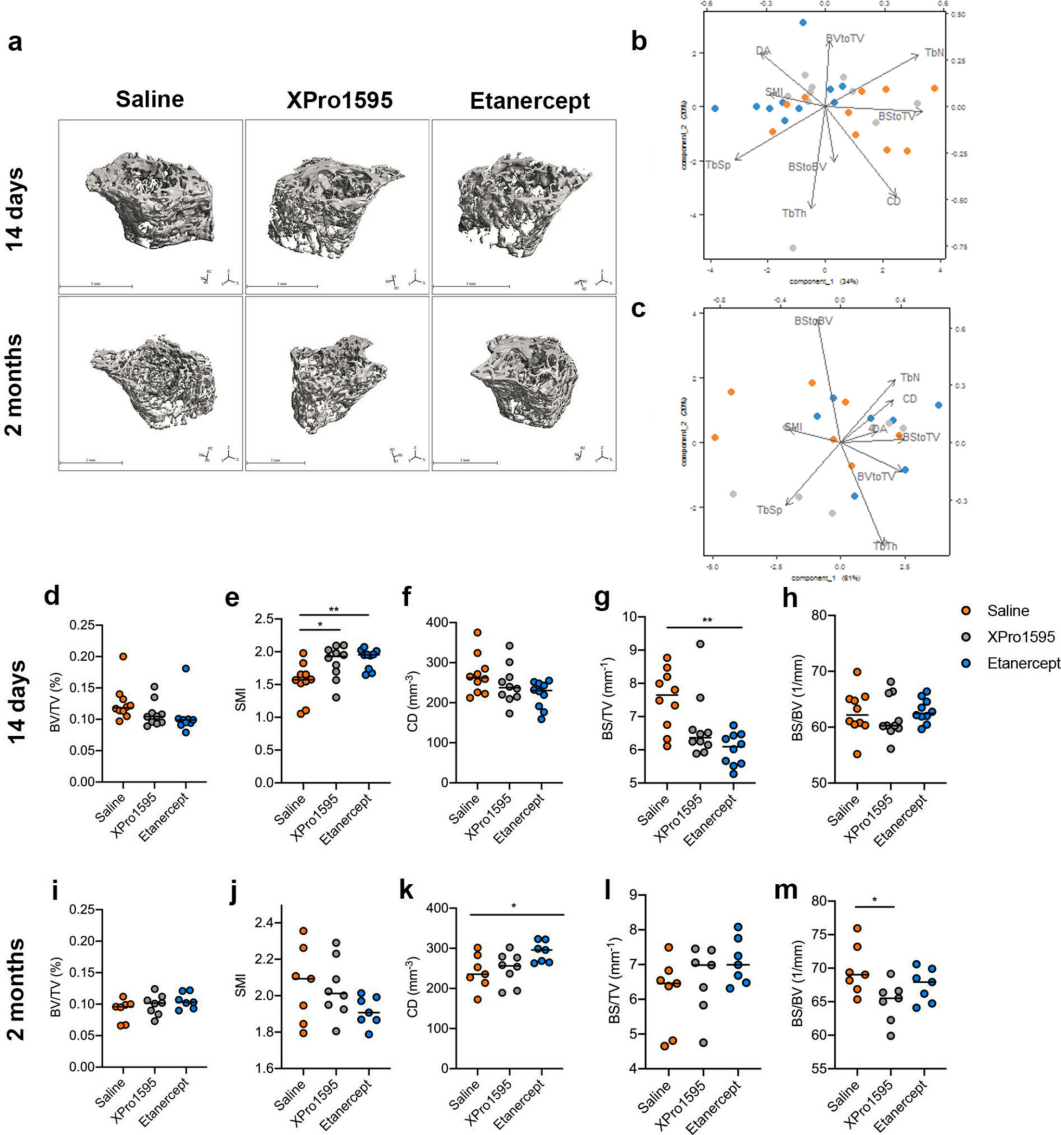
Effect on bone microstructure of anti-TNF treatment. (**a**) Representative micro-CT images of humeri from mice treated for 14 days, and 2 months with saline, XPro1595, or etanercept. (**b,c**) PCA analysis of trabecular bone morphology following 14 days (**b**) and 2 months (**c**) saline, XPro1595, or etanercept treatment. (**d-h**) Bone volume fraction (BV/TV) (%) (**d**) structure model index (SMI) (**e**) connectivity density (CD) (mm^-3^) (**f**) bone surface density (BS/TV) (mm^-1^) (**g**) and bone surface to volume ratio (BS/BV) (mm^-1^) (**h**) measurements 14 days after saline, XPro1595, or etanercept treatment. (**i-m**) BV/TV (%) (**i**) SMI (**j**) CD (mm^-3^) (**k**) BS/TV (mm^-1^) (**l**) and BS/BV (mm^-1^) (**m**) measurements 2 months after saline, XPro1595, or etanercept treatment. One-way ANOVA, Tukey’s post hoc, *n* = 7–10/group, *P < 0.05, **P < 0.00 2.

Next, we assessed whether 14 days and 2 months of XPro1595 and etanercept treatment affected trabecular bone morphology using high-resolution micro-CT (Figure 2 and Table 2). We scanned the trabecular bone of the proximal humeri in healthy mice treated with saline, XPro1595, or etanercept 14 days or 2 months after the start of treatment (Figure 2a).

**Table 2:** Comparison of microarchitectural properties of mouse humoral cancellous bone between groups (mean ±SD)

Microarchitectural properties	Bone volume fraction (%)	Structure model index (-)	Trabecular thickness (μm)	Degree of anisotropy (-)	Connectivity density (mm^-3^)	Bone surface density (mm^-1^)	Bone surface to volume ratio (mm^-1^)	Trabecular separation (μm)	Trabecular number (mm^-1^)
14 Days									
G1: Saline	0.13 ± 0.03	1.55 ± 0.28	0.040 ± 0.003	1.80 ± 0.19	269.2 ± 49.8	7.53 ± 0.90	62.62 ± 3.98	0.30 ± 0.04	3.47 ± 0.47
G2: XPro1595	0.11 ± 0.02	1.84 ± 0.26	0.041 ± 0.003	1.83 ± 0.15	245.2 ± 48.4	6.68 ± 1.00	61.77 ± 3.79	0.29 ± 0.03	3.53 ± 0.33
G3: Etanercept	0.10 ± 0.03	1.90 ± 0.15	0.040 ± 0.002	1.95 ± 0.10	220.4 ± 33.7	6.02 ± 0.49	62.90 ± 2.14	0.32 ± 0.04	3.27 ± 0.40
One-way ANOVA	*P*=0.18	*P*=0.006	*P*=0.76	*P*=0.10	*P*=0.07	*P*=0.002	*P*=0.74	*P*=0.32	*P*=0.32
Difference btw groups		G1 < G2,G3				G1 > G3			
2 months									
G1: Saline	0.09 ± 0.02	2.06 ± 0.21	0.035 ± 0.003	1.69 ± 0.08	240.9 ± 43.2	6.17 ± 1.05	69.70 ± 3.68	0.25 ± 0.02	4.00 ± 0.28
G2: XPro1595	0.10 ± 0.02	2.04 ± 0.16	0.039 ± 0.003	1.72 ± 0.10	248.8 ± 40.2	6.55 ± 0.98	64.97 ± 3.04	0.25 ± 0.03	4.00 ± 0.42
G3: Etanercept	0.11 ± 0.01	1.92 ± 0.08	0.037 ± 0.002	1.76 ± 0.07	290.9 ± 26.1	7.08 ± 0.66	67.42 ± 2.50	0.24 ± 0.02	4.20 ± 0.38
One-way ANOVA	*P*=0.23	*P*=0.21	*P*=0.12	*P*=0.26	*P*<0.05	*P*=0.20	*P*=0.04	*P*=0.67	*P*=0.69
Difference btw groups					-		G1 > G2		

The PCA biplot revealed that 14-day etanercept-treated mice did not exhibit changes in major parameters of cancellous bone microarchitecture, such as bone volume fraction and TbTh, although some alternations in microarchitecture were observed (Figure 2b, Table 2).

Neither 14-day (Figure 2d) nor 2-month (Figure 2i) XPro1595 or etanercept treatment affected BV/TV. The SMI significantly increased in 14-day XPro1595-treated mice (*P*<0.05) and even more so in 14-day etanercept-treated mice (*P*<0.01) (Figure 2e), whereas 2-month treatment had no effect on SMI (Figure 2j). The CD was unaffected by 14-day treatment (Figure 2f) but significantly increased following 2-month treatment (one-way ANOVA *P*<0.05, post hoc Tukey’s test non-significant) (Figure 2k). The BS/TV significantly decreased in mice with 14-day etanercept treatment compared with saline-treated mice (*P*<0.01) (Figure 2g) but was unaffected by 2-month XPro1595 or etanercept treatment (Figure 2l). The BS/BV ratio was unaffected following 14-day XPro1595 or etanercept treatment (Figure 2h), but significantly decreased in 2-month XPro1595-treated mice compared with saline-treated mice (*P*<0.05) (Figure 2m). TbTh, DA, TbSp, and TbNs were unaffected by both 14-day and 2-month of XPro1595 or etanercept treatment (Table 2).

### XPro1595 and etanercept treatments do not significantly affect functional outcome five days after experimental supraspinatus tendon tear

In order to investigate the potential of anti-TNF therapy on muscle inflammation after experimental supraspinatus tendon tear, we subjected C57BL/6 mice to experimental supraspinatus tendon tear followed by either XPro1595, etanercept, or saline treatment and let the mice survive five days post-injury.

**Figure 3:**
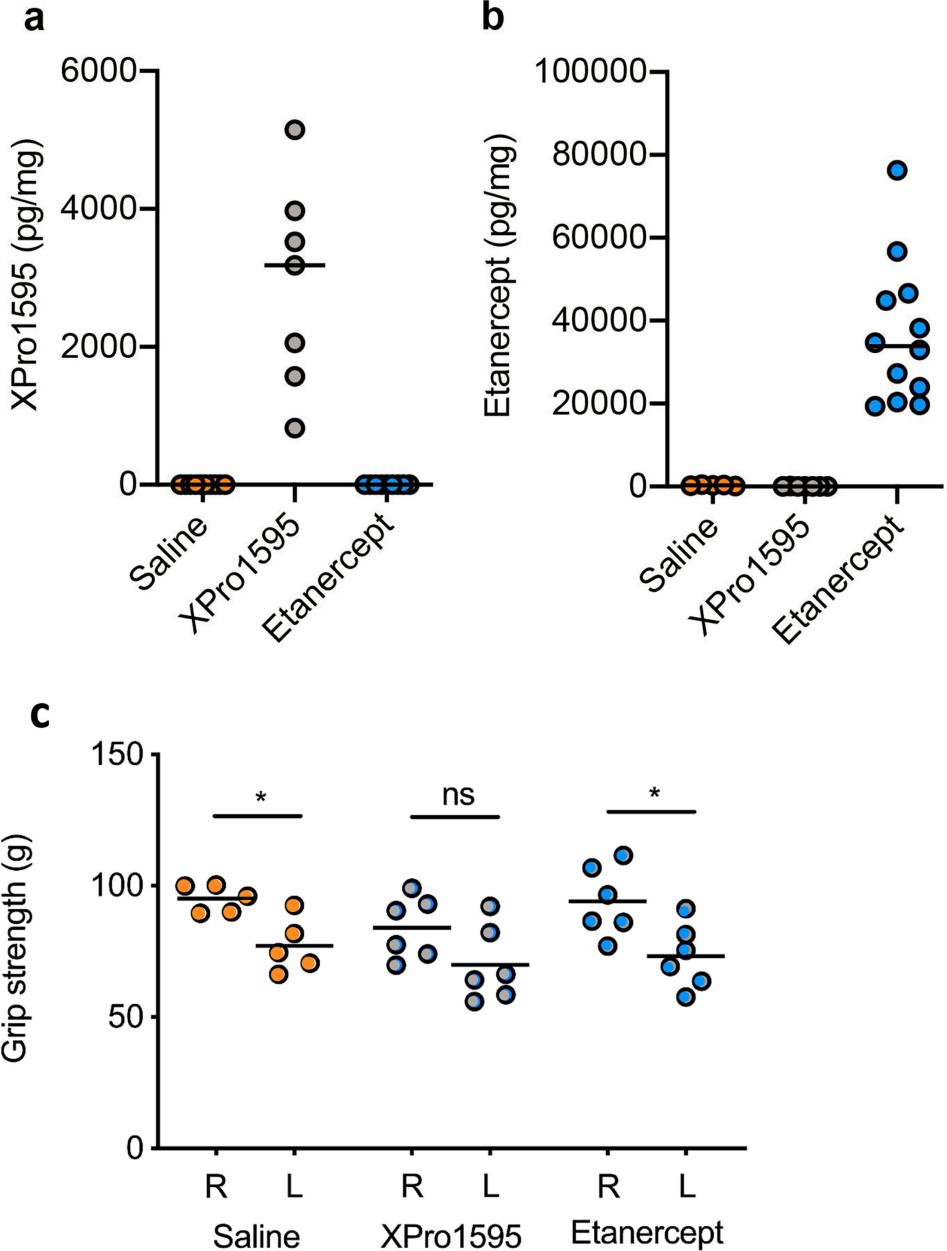
Concentrations of TNF inhibitors in supraspinatus muscle tissue and grip strength five days after experimental supraspinatus tendon tear. (**a**) Concentration of XPro1595 in supraspinatus muscle tissue five days after supraspinatus tendon tear. (**b**) Concentration of etanercept in supraspinatus muscle tissue five days after supraspinatus tendon tear. *n* = 10–12 supraspinatus muscles/group. XPro1595 and etanercept concentrations were measured by chemiluminescence in both lesioned and non-lesioned supraspinatus muscles. (**c**) Grip strength in the lesioned, left (L) and non-lesioned, right (R) fore limb.*saline *P*=0.03, *etanercept *P*=0.01.

Both XPro1595 (Figure 3a) and etanercept (Figure 3b) were found to successfully penetrate supraspinatus muscle tissue in relevant therapeutic doses [25] after supraspinatus tendon tear.

In line with our DEXA results, we observed that neither XPro1595 nor etanercept affected weight changes after experimental supraspinatus tendon tear (Δweight change: Saline 0.55 g ± 0.44 g, XPro1595 0.10 g ± 0.41 g, and etanercept 0.91 g ± 0.80 g, *n* = 6/group, *P*=0.08).

Grip strength (Figure 3c) was significantly decreased on the lesioned front limb compared with the non-lesioned front limb in saline-treated (lesioned: 95.2 g ± 5.2 g, non-lesioned: 77.2 g ± 10.3 g, paired Student’s t-test *P*=0.03) and etanercept-treated mice (lesioned: 94.1 g ± 13.3 g and non-lesioned: 73.1 g ± 12.2 g, *P*=0.01), but not significantly decreased on the lesioned front limb in XPro1595-treated mice (lesioned: 84.0 g ± 11.8 g and non-lesioned: 69.9 g ± 14.3 g, *P*=0.06), which could indicate a protective effect of XPro1595. However, there was no significant difference between the effect of saline, etanercept, and XPro 1595 (Kruskal–Wallis test *P*=0.91).

### The main differences between lesioned and non-lesioned supraspinatus muscles relate to muscle cytoskeleton and extracellular matrix

**Figure 4:**
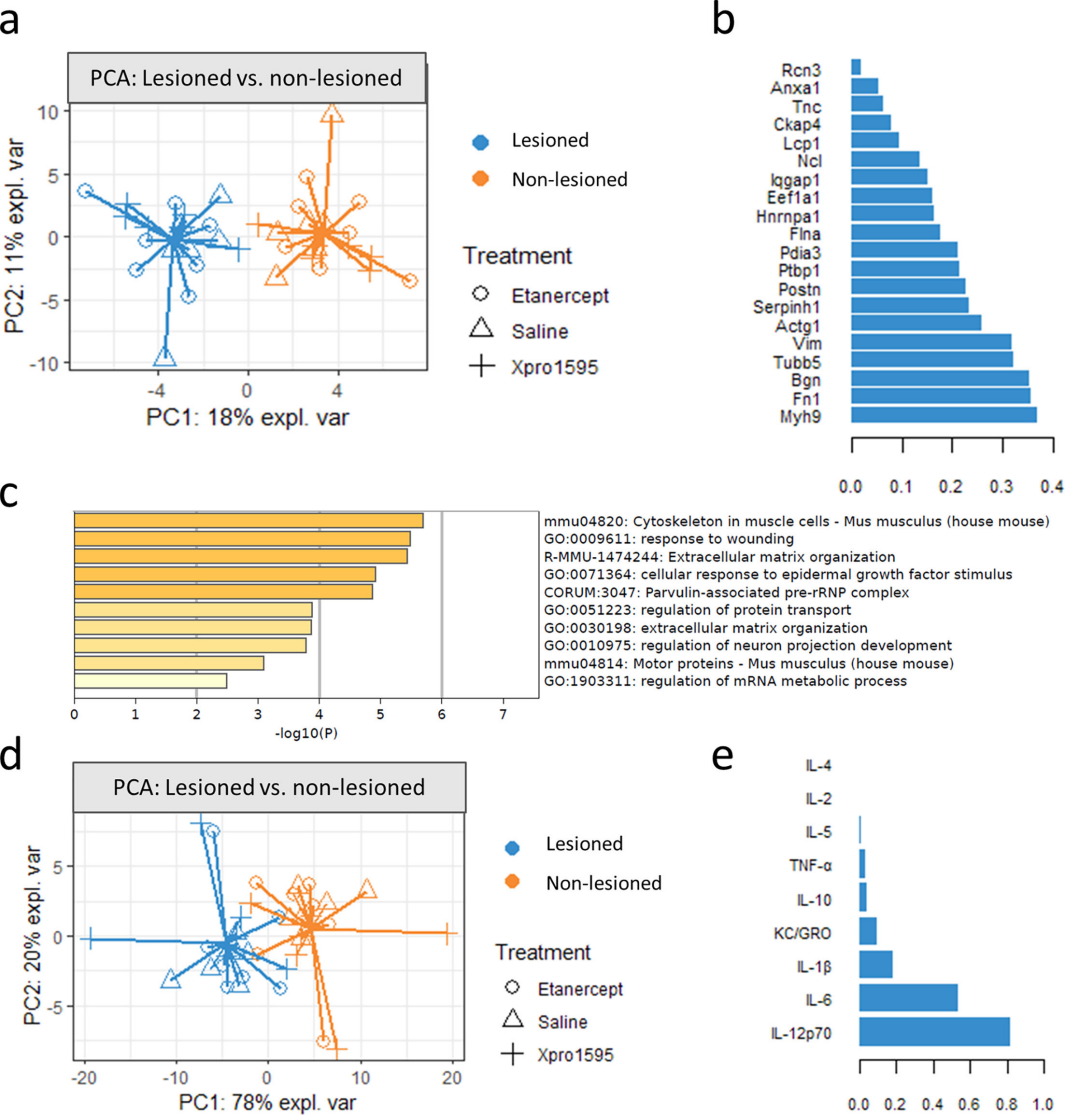
Analyses of supraspinatus muscle proteome and cytokine differences between lesioned and non-lesioned sides**.** (**a**) Principal component analysis showed a perfect clustering of supraspinatus muscle proteome according to lesioned and non-lesioned side. (**b**) Loadings of the 20 proteins on component 1 that accounts for the major source of variation in the supraspinatus muscle proteome, i.e. proteins that were up-regulated in lesioned muscle compared with non-lesioned supraspinatus muscle. (**c**) A Metascape analysis of the 20 proteins that were up-regulated in the lesioned supraspinatus muscle showed multiple protein–protein interactions related to the cytoskeleton in muscle cells, response to wounding, and extracellular matrix organization (**d**) Principal component analysis based on cytokine data also clustered according to the lesioned and non-lesioned side. (**e**) Loadings of cytokines on component 1 showed that IL-12p70, IL-6, and IL-1β were the most up-regulated cytokines in the lesioned muscle.

There was a clear separation of the non-lesioned and lesioned muscle proteomes in the multilevel PCA model, meaning that the effect of surgery was the biggest source of proteomic variation between samples (Figure 4a). The 20 most important proteins for discriminating between lesioned and non-lesioned proteomes are listed in Figure 4b. A metascape analysis [26] of these 20 proteins revealed significant protein–protein interactions related to the cytoskeleton in muscle cells, response to wounding, and extracellular matrix organization, among others (Figure 4c).

A clear separation of the non-lesioned and lesioned sides was likewise observed on the PCA model that was based on cytokine data (Figure 4d). Cytokines that gave rise to the most variability between non-lesioned and lesioned muscles were IL-12p70, IL-6, and IL-1β (Figure 4e).

### XPro1595 and etanercept treatment does not affect cytokine levels five days after supraspinatus tendon tear but has a minimal impact on the proteome

**Figure 5:**
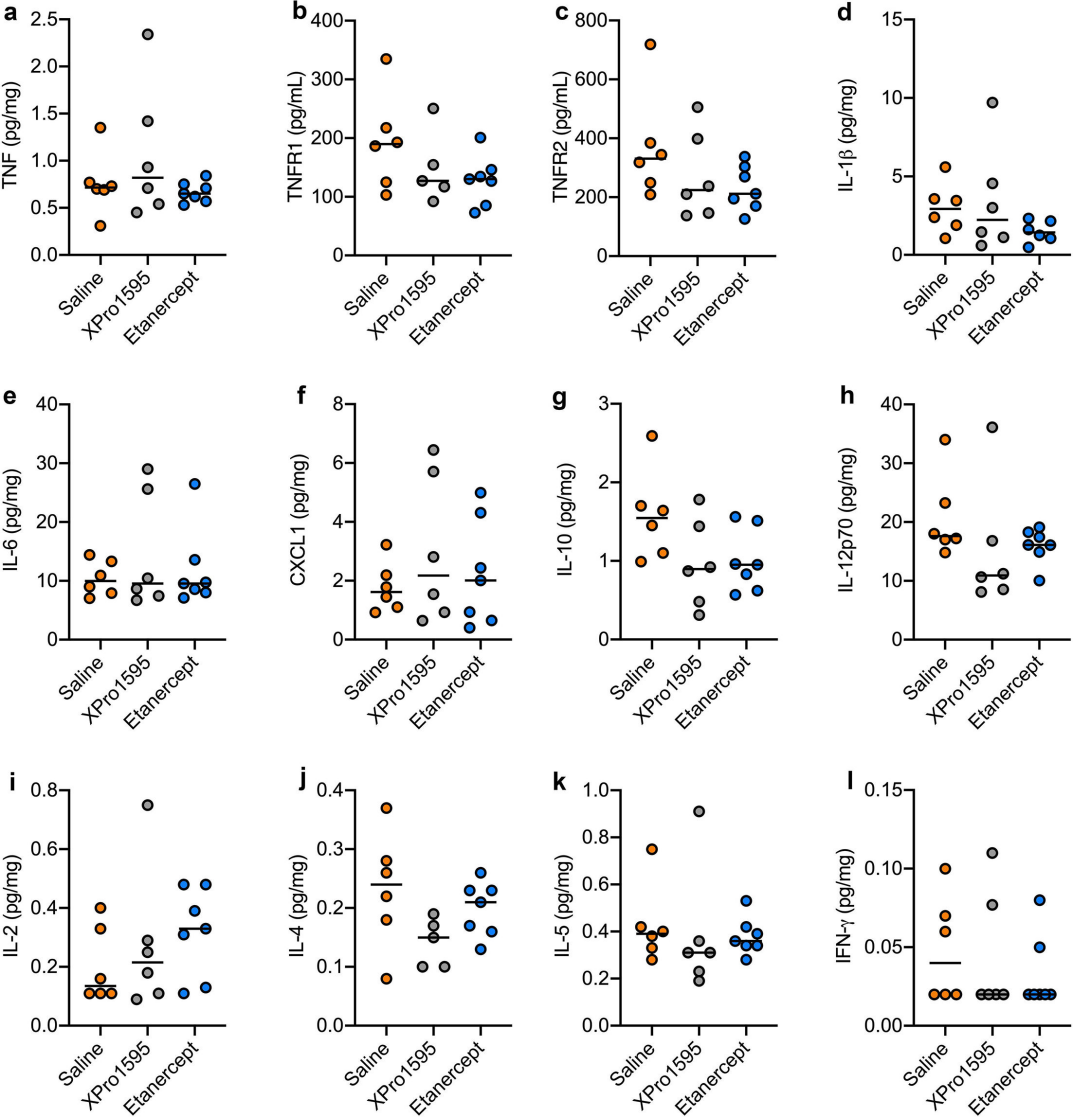
Cytokine analysis of lesioned supraspinatus muscle tissue five days after experimental supraspinatus tendon tear. (**a-l**) Electrochemiluminescence analysis of lesioned supraspinatus muscle tissue five days after experimental supraspinatus tendon tear demonstrated no significant changes in TNF (*P*=0.28) (**a**) TNFR1 (*P*=0.20) (**b**) TNFR2 (*P*=0.22) (**c**) IL-1β (*P*=0.31) (**d**) IL-6 (*P*=0.54) (**e**) CXCL1 (*P*=0.68) (**f**) IL-10 (*P*=0.09) (**g**) IL-12p70 (*P*=0.98) (**h**) IL-2 (*P*=0.68) (**i**) IL-4 (*P*=0.12) (**j**) IL-5 (*P*=0.12) (**k**) or IFN-γ (*P*=0.67) (**l**) protein levels. One-way ANOVA, *n*=6-7/group.

In order to investigate whether XPro1595 or etanercept affected cytokine and TNF receptor levels in the lesioned supraspinatus muscle, we performed electrochemiluminescence analysis for TNF (Figure 5a), TNFR1 (Figure 5b), TNFR2 (Figure 5c), IL-1β (Figure 5d), IL-6 (Figure 5e), CXCL1 (Figure 5f), IL-10 (Figure 5g), IL-12p70 (Figure 5h), IL-2 (Figure 5i), IL-4 (Figure 5j), IL-5 (Figure 5k), and IFN-γ (Figure 5l). Neither XPro1595 nor etanercept treatment, however, significantly affected the levels of cytokine, TNFR1, or TNFR2 compared with saline.

**Figure 6:**
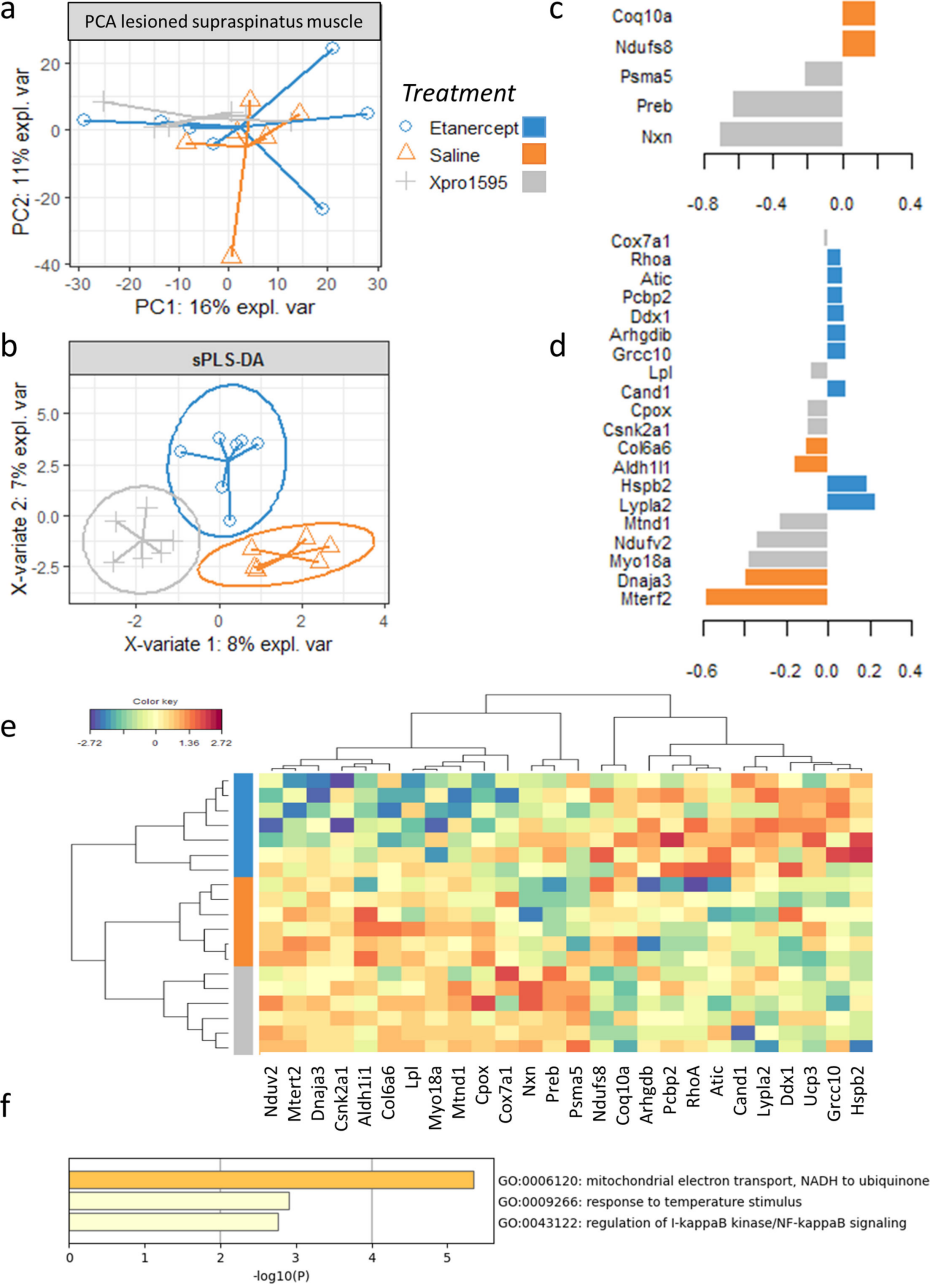
Effect of treatment on the proteome. (**a**) The supraspinatus muscle proteome did not cluster naturally according to treatment in the principal component analysis (PCA). (**b**) The sparse partial least squares discriminant analysis (supervised model) was able to find proteins for discrimination of the three treatment groups. (**c**) 5 proteins that explained the proteomic difference between Xpro1595 and saline-treated mice. Colors of the bars indicate which group had the highest expression of a particular protein. (**d**) 20 proteins explain the proteomic difference between etanercept versus Xpro1595 and saline-treated mice. Colors of the bars indicate which group had the highest expression of a particular protein. (**e**) Heat map with clustering based on the proteins that discriminated mice according to the treatment they received. The etanercept group appeared to be different from the two other groups based on their protein profile, and the difference between the Xpro1595 and saline groups was not visually apparent by means of relative protein abundance. (**f**) A functional enrichment analysis revealed that the proteins responsible for discrimination of the etanercept group from the Xpro1595 and saline groups were related to mitochondrial electron transport, NADH to ubiquinone, response to temperature stimulus and regulation of I-κB kinase /NF-κB signaling.

Proteomes from the lesioned side did not cluster together according to treatment in the PCA model, meaning that the effect of treatment on the proteomes is not large enough to be detected in an unsupervised manner (Figure 6a). However, the sPLS–DA was able to identify predictive variables for discriminating between proteomes according to treatment, as indicated by the sPLS–DA score plot (Figure 6b). The most important proteins for discriminating between the three treatment groups are listed in (Figure 6e-d). Xpro1595 differed from etanercept and saline by up-regulation of three proteins. In muscle tissue, nucleoredoxin (nxn) is expressed mainly in endothelial cells, but also in fibroadipogenic progenitor cells (FAP) and myofibers [27]. It is a negative regulator of the Wnt signaling pathway. Prolactin regulatory element binding (preb) is a transcription factor that regulates prolactin gene expression, is also able to activate MCP-1, a major chemotactic factor for monocytes and a key factor initiating the inflammatory process of atherogenesis. Proteasome 20S subunit alpha 5 (psma5) is a component of the 20S core proteasome complex involved in the proteolytic degradation of most intracellular proteins. Both preb and psma5 are expressed in all cell types in skeletal muscle tissue [27]. The three up-regulated proteins, however, did not participate in any common gene ontology.

The clustered image map, which was subsequently made using the proteins with highest impact for discriminating the three treatment groups based on the proteome profile of the lesioned supraspinatus muscle, showed a perfect clustering of samples corresponding to the treatment (Figure 6b and e). The difference between the etanercept and saline groups appeared to be more different than the difference between the Xpro1595 and saline groups as evidenced by the dendrogram (Figure 6e). In fact, the saline and Xpro1595 groups appeared to have similar protein profiles, but the etanercept group was different from the saline and Xpro1595 groups. This difference was explained by a down-regulation of Ndufv2, Mterf2, Dnaja3, Csnk2a1, Aldh1l1, Col6a6, Lpl, Myo18a, Mtnd1, Cpox, Cox7a1, Nxn, Preb, and Psma5 and an up-regulation of NADH:ubiquinone oxidoreductase core subunit S8 (Ndufs8), Coenzyme Q10A (Coq10a), Arhgdib, Pcbp2, Rhoa, Atic, Cand1, Lypla2, Ddx1, Ucp3, Grcc10, and Hspb2 in the etanercept group compared with the Xpro1595 and saline groups. A functional enrichment analysis revealed that these proteins were related to mitochondrial electron transport, NADH to ubiquinone, response to temperature stimulus, and regulation of I-κB kinase/NF-κB signaling (Figure 6f), suggesting that these biological processes are differentially regulated in the etanercept group compared with the Xpro1595 and saline groups.

### Quantitative histological analyses of supraspinatus muscle tissue

**Figure 7:**
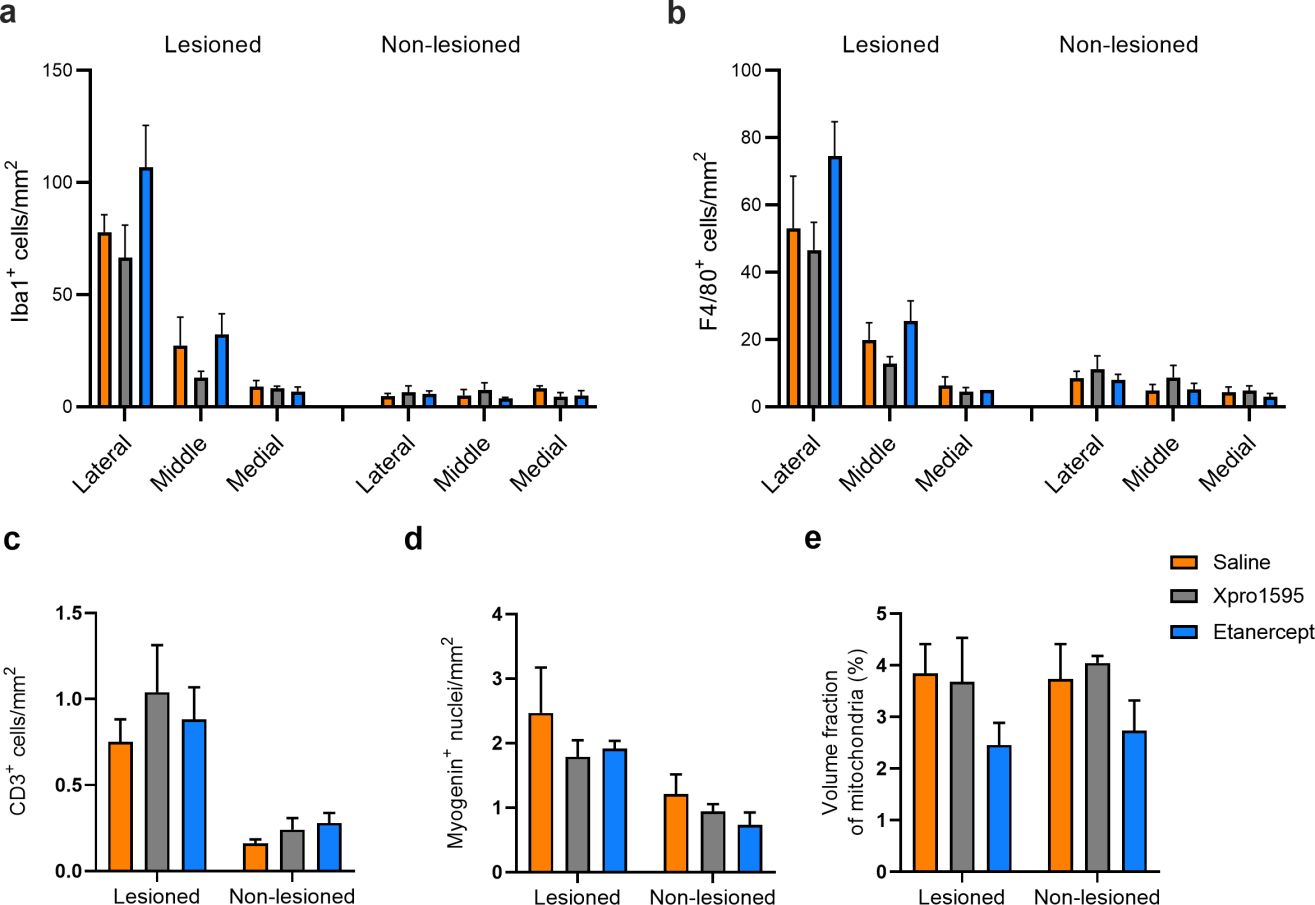
Density of macrophages, T-cells, myogenin-positive nuclei, and volume fraction of mitochondria in murine supraspinatus muscles five days after experimental tendon tear are shown as mean with SEM. In a and b, the density of macrophages was determined by the macrophage markers Iba1 and F4/80 respectively. Positive cells were counted in the tendon near the lateral end, in the middle of the muscle, and in the medial end furthest away from the tendon, respectively. In c and d, the number of positive cells or nuclei was counted in the entire supraspinatus sections. In e, the volume fraction of mitochondria was estimated using immunofluorescence of the mitochondrial marker Tom20. *n*=5 mice per group for XPro1595 and etanercept, and *n*=4 in the saline-treated group. One mouse in this group was excluded because of a high degree of inflammation in the lesioned muscle.

Histomorphometric analyses of the inflammatory response to tendon lesion in the supraspinatus muscle showed increased presence of both macrophages and T-lymphocytes (*P*<0.0001 for all markers) in the lesioned muscle compared with the non-lesioned muscle (Figure 7a-c). The increase in macrophage density was evident and allowed analyses of regional variation. These demonstrated a significantly higher content of macrophages in the most lateral third of the muscle, closest to the tendon, as compared with the more medial regions of the muscle (*P*<0.0001 for the lesioned muscle and *P*=0.0496 for the non-lesioned muscle).

Myogenin (Figure 7d), an indicator of muscle regeneration, likewise increased in the lesioned muscle sections (*P*=0.0002). The volume fraction of subsarcolemmal mitochondria density in TOM-stained sections showed no side differences (Figure 7e).

Concerning the effect of anti-TNF treatment in the lesioned supraspinatus muscles, the tendencies were that XPro1595-treated mice had a lower macrophage density than saline, while etanercept-treated mice had a higher macrophage density compared with saline (Figure 7a). This applied to both macrophage markers, Iba1 and F4/80. The same tendencies were not found concerning T lymphocytes or myogenin expression (Figure 7e-d). Mitochondrial density appeared lower bilaterally in the etanercept-treated animals (Figure 7e), but the difference did not quite reach statistical significance (*P*=0.07).

## Discussion

Acute inflammation is a vital and immediate response to tissue trauma, as it initiates tissue repair [16]. The inflammatory processes are regulated by the innate immune system and respond to restore the structurally damaged tissues. Persistent dysregulated inflammation, however, has been shown in RC tissues in relation to tendon tear. These inflammatory processes, in particular, may lead to continued cell and tissue malfunction and therefore are a target for regulation. The judicious application of anti-inflammatory drugs applied in chronic inflammatory diseases could therefore be beneficial in combination with RC repair.

Based on this background, we have studied the effect of selective and non-selective anti-TNF treatment on musculoskeletal tissues around the shoulder. To detect possible side effects, we also analyzed the influence on body and bone composition.

### Anti-TNF treatment does not affect body composition but has a slight influence on bone composition

Though the major side effects of anti-TNF treatment reported are related to the immune system, there may be some metabolic effects as well. TNF can dysregulate adipocyte biology [28,29]. The effects on bone depend on the local environment, thus TNF can stimulate osteoblast reabsorption of bone and inhibit bone formation and matrix production [30]. However, in certain inflammatory situations, an osteogenic effect has been observed.

Anti-TNF treatment did not affect body weight nor body composition, including overall bone mineral density and content, and bone area based on DEXA. However, when the detailed microstructure of the humerus obtained with micro-CT was analyzed, the cancellous bone microstructure revealed changes that could indicate that etanercept induced slight initial decrease and a later increase in cancellous bone, a possible effect of osteoclast inhibition and osteoblast stimulation. The XPro1595 effect on bone at 2 months was only seen in the bone CD and BS/BV.

### Supraspinatus tendon tear-induced changes in the supraspinatus muscles

We have previously studied the effect of supraspinatus tendon lesion on the supraspinatus muscle in mice and RC patients by proteomics. We found that pathways concerning extracellular matrix, inflammation, and muscle structure were influenced in the lesioned muscles [16,31]. The present proteome analyzes show changes in the same fields. In our previous flow cytometric analyses [16], we found signs of inflammation in the lesioned side but no up-regulation of T lymphocytes. However, the number of T lymphocytes was significantly up-regulated in the lesioned side in our present digital quantitation on sections. The macrophage response is the major inflammatory response, and a detailed analysis of the distribution of macrophages in the supraspinatus muscle showed a significantly higher macrophage density in the tendon-near zone compared with the rest of the muscle. This was found on both sides but was most pronounced in the lesioned muscles. The zonal distribution also in the non-lesioned muscle suggests that the function of macrophages in the normal maintenance of the muscle in the myotendinous zone is augmented by lesions.

Counting on sections also indicated a higher myogenic activity on the lesioned side seen as a significant increase in nuclei expressing the late myogenic factor myogenin.

We found no significant differences in the cytokine levels, although the proinflammatory IL-12p70, IL-6, and IL-1β all tended to be up-regulated at the lesioned site. This is in line with our previous findings [16] where IL-6 and IL-1β significantly differed between lesioned and nonlesioned sides.

### Etanercept treatment differs more from saline than XPro1595 treatment does

TNF has been shown to be involved in the development of muscle atrophy [4]. Studies on inhibition of TNF with infliximab have shown inhibition of inflammation and reduced apoptosis in a rat model for traumatized skeletal muscles [32]. Also, in a rat model for RC injury, etanercept treatment was described to reduce inflammation, apoptosis, and fibrosis [33].

Our analysis of muscle tissue on the lesioned side demonstrated that both TNF inhibitors were present in the muscle after i.p. injections, but only a few proteins were differentially expressed between saline- and XPro1595-treated animals. The mitochondrial proteins ndufs8 and coq10a were both down-regulated compared with saline, suggesting an influence on mitochondrial function.

In etanercept-treated mice, ten proteins were down-regulated, and ten proteins were up-regulated, compared with saline and XPro1595. Among the down-regulated proteins, five were related to mitochondria. An involvement of mitochondria was supported by the tendency to a reduced volume of mitochondria in muscle fibers in etanercept-treated muscles found in the histology.

The difference between etanercept and XPro1595 could be related to XPro1595 blocking only solTNF.

### Proteomics suggests reduced apoptosis and antifibrosis in etanercept compared with XPro1595 and saline-treated mice

Since high numbers of mitochondrial transcripts are associated with apoptosis [34], the lower mitochondrial protein expression in etanercept-treated mice could reflect a protective effect against apoptosis as seen in treatment with TNF inhibitors in rats [32,33]. Also, the difference in NF-κB signaling gene ontology by Rhoa, Dnaja3, Ddx1, Csnk2a1, and Arhgdib could support an influence on apoptosis [4]. The reduced col6a6 in the etanercept-treated mice could indicate an antifibrotic effect.

## Conclusion

Our study demonstrates that lesions of the supraspinatus tendon provoke an inflammatory response with a distinct topographical pattern, as well as the activation of a myogenic response. With regard to TNF inhibition, our proteomic analysis reveals that mitochondrial proteins are significantly affected, consistent with the known anti-apoptotic effects. Additionally, we observe that the effects of blocking solTNF using XPro1595 are less pronounced compared with those of the non-selective TNF inhibitor etanercept. However, in this model, the effect of TNF blockade on inflammation was not effective and thus cannot be recommended to treat RC injuries.

## Data Availability

Data are available via ProteomeXchange with identifier PXD035331 (Data in support of TNF inhibition in experimental rotator cuff tear mouse model) (35, 36) at Website: http://www.ebi.ac.uk/prideReviewer account details: Username: reviewer_pxd035331@ebi.ac.uk; Password: HxnQSiG0

## Abbreviations

ACN: acetonitrile
BMC: bone mineral content
BMD: bone mineral density
BS/BV: bone surface to volume ratio
BS/TV: bone surface density
BV/TV: bone volume fraction
CD: connectivity density
DA: degree of anisotropy
DEXA: dual-energy X-ray absorptiometry
FA: formic acid
HRP: horseradish peroxidase
IFN-γ: interferon-gamma
IL: interleukin
LC: liquid chromatography
LLOD: lower limit of detection
MS: mass spectrometry
PCA: principal component analysis
PFA: paraformaldehyde
RC: rotator cuff
SMI: structure model index
SS: Supraspinatus
TFA: trifluoroacetic acid
TNF: tumor necrosis factor
TOM: translocase of the outer membrane
TbN: trabecular number
TbSp: trabecular separation
TbTh: trabecular thickness
coq10a: coenzyme Q10A
i.p.: intraperitoneally
ndufs8: NADH ubiquinone oxidoreductase core subunit S8
nxn: nucleoredoxin
preb: prolactin regulatory element binding
psma5: proteasome 20S subunit alpha 5
sPLS–DA: sparse partial least squares–discriminant analysis
s.c.: subcutaneously
solTNF: soluble form of TNF
